# Association of workplace bullying and burnout with nurses’ suicidal ideation in Bangladesh

**DOI:** 10.1038/s41598-023-41594-4

**Published:** 2023-09-05

**Authors:** Humayun Kabir, Saifur Rahman Chowdhury, Anjan Kumar Roy, Samiul Amin Chowdhury, Md. Nazrul Islam, Rifat Jahan Chomon, Masuda Akter, Ahmed Hossain

**Affiliations:** 1 Department of Public Health, North South University, Dhaka, 1229 Bangladesh; 2 Department of Health Research Methods, Evidence and Impact (HEI), McMaster University, 1280 Main Street West, Hamilton, ON L8S 4L8 Canada; 3 Department of Nursing and Health Science, Jashore University of Science and Technology, Jashore, 7408 Bangladesh; 4 Department of Public Health, Leading University, Sylhet, 3112 Bangladesh; 5 Department of Community Health and Epidemiology, College of Medicine, University of Saskatchewan, Saskatoon, SK S7N 5E5 Canada; 6 Faculty of Medicine, University of Dhaka, Dhaka, 1000 Bangladesh; 7 Health Services Administration, College of Health Sciences, University of Sharjah, Sharjah, UAE; 8 Global Health Institute, North South University, Dhaka, 1229 Bangladesh

**Keywords:** Public health, Epidemiology, Risk factors

## Abstract

Suicidal ideation is a complex phenomenon influenced by several predisposing, contextual, and mediating factors that seem more common among healthcare workers, especially nurses. We investigated the association of bullying and burnout with suicidal ideation among Bangladeshi nurses and identified the associated factors. We conducted a cross-sectional study among 1264 nurses in Bangladesh between February 2021 and July 2021. We applied a modified Poisson regression model with robust error variance to determine the association of bullying and burnout with suicidal ideation. Among 1264 nurses, the female was 882 (70.02%), and the mean age was 28.41 (SD = 5.54) years. The prevalence of high levels of suicidal ideation was 13.26%. In the Poison regression model, high risk bullying (RR = 6.22, 95% CI 3.13–12.38), targeted to bullying (RR = 7.61, 95% CI 3.53–16.38), and burnout (RR = 8.95, 95% CI 2.84–28.20) were found to be significantly associated with suicidal ideation. Furthermore, we found significant interaction between workplace bullying and burnout with suicidal ideation (*p* < 0.05). The high prevalence of bullying, burnout, suicidal ideation, and their association indicate an unsafe workplace for the nurses. Initiatives are needed to make a favorable work environment to improve nurses' overall mental health and reduce suicide ideation.

## Introduction

Suicide is a major global problem that the global community is struggling to prevent; however, the prevalence of suicide is still sky-rocking. There are numerous causes of death, but suicide claims over 700,000 lives each year, accounting for 1.4% of all deaths worldwide^1^. Suicidal ideation is a complex phenomenon that is influenced due to several predisposing, contextual, and mediating factors^2^. For example, among healthcare workers, an individual's life or work-related issues may worsen their mental health, and that may lead to suicide^3^. Preventing the rising trend of suicide requires a unique global initiative to be taken by the stakeholders.

Suicidal ideation is known as having thoughts, ideas, or ruminations that take place before committing suicide and attempting suicide^4^. The likelihood of suicide attempts increases due to the higher level of suicidal ideation for a more extended period^5^. Nurses are one of the most prevalent in suicide among healthcare workers; previous study observed their lacking knowledge about suicide prevention^6^. The suicide trend was noticeably two times higher among nurses than the general population in the United States^5^. Regular screening and research for suicide precursors, on the other hand, can assist in determining those who are at high risk of suicide, and proper measures can help reduce the rising rate of suicide among nurses^7^. A study in Bangladesh found that one out of every ten nurses in a tertiary care hospital had suicidal thoughts at some point in their lives^8^. Suicidality among nurses is influenced by a variety of factors, including long work hours, being the primary caregiver and working in the critical care area, and experiencing workplace bullying and burnout^9^.

The interpersonal theory of suicide (IPTS) suggests that workplace bullying can contribute to suicidal thoughts through two key factors: “thwarted belongingness” (feeling socially isolated) and “perceived burdensomeness” (believing one is a burden on others with little hope of improvement)^10,11^. Bullying has been reported to be associated with nurses’ suicidal ideation, suicidal attempts, and their suicide^12^. Numerous studies reported workplace bullying among nurses, which occurs primarily as a result of the authority's aggressive, violent, and frightening behavior or insulting behaviours by colleagues that make them feel irritated, humiliated, and threatened; thereby, their mental health is supposed to have deteriorated^13,14^. According to a study, bullying among nurses is a fairly common occurrence that harms both nurses and patients^12^. It has been reported that the risk of patient death in clinical settings is significantly associated with bullying against nurses^15^. According to a study in the United States, more than half of nurses reported that bullying occurs frequently, and it occurs mostly during their working time^16^. Another study in the Pacific Northwest state (Alaska) of the United States found that 28% of registered nurses reported being bullied daily, while almost one-third reported being bullied every week^17^. The statistics are even more startling for new nurses, with more than 72% reporting bullying in the previous month, according to research conducted in the state of Ohio, Kentucky, and Indiana in the United States^18^.

Burnout is a state of fatigue related to emotional and depersonalization, also often a decrease in personal accomplishments, which results in unmanaged work-related stress^19^. The findings of Pompili et al.^20^ suggested that burnout and certain defence mechanisms were predictors of hopelessness, which was then a significant indicator of suicide risk. Privious study found that burnout is a significant predictor of nurse suicidal ideation and suicide^21^. Burnout among healthcare workers is a common mental health phenomenon that is caused by numerous work-related factors^19,22, 23^. It appears to be more prevalent among doctors, nurses, and social workers^24^. A meta-analysis found that the global prevalence of burnout among nurses is 11.23%^25^. Additionally, a cross-sectional study in Bangladesh reported that most ICU personnel, especially nurses, experience burnout to some extent^26^. Chin et al.^27^ conducted a study in Taiwan, which reported that higher levels of burnout and perpetual work stress are the main contributors to nurses considering suicide ideation. A study in the United States found a strong association between burnout and suicidal ideation, with 5.5% of nurses reporting suicidal ideation because of burnout^28^. The clinical nurses' burnout is also correlated with workplace bullying, compromising the standard of nursing care and patient safety^29^.

In Bangladesh, no study previously investigated suicidal ideation and its associated factors among nurses, although this association was established in several countries, as discussed above. In Bangladesh, nursing is predominantly considered a female profession and is often perceived as having lower status compared to other medical professions, both from a societal and economic standpoint. For instance, within the government scale, nurses are typically placed in the second-class grade (10th) at the start of their careers, while positions such as physicians and dentists begin at the first-class grade (9th) or higher^30^. This dominance by other professions or the segregation in payment structure in nursing further reflects the overall societal, policymaker’s, and government’s attitude toward nurses in Bangladesh, which is influenced by the dominance of other professions who are in power; maybe they are men in the majority. Hadley et al.^31^ reported more detail on the contextual difference between the British model of nursing and the Bangladesh model of nursing.

The contextual disparity in Bangladesh regarding the nursing profession warrants further research due to its potential to strain nurses' mental health and increase the risk of suicidal ideation. Moreover, previous studies found that nurses in Bangladesh encounter higher level of depression, anxiety, and stress^32^. We designed the current study to explore the suicidal ideation among Bangladeshi nurses and the association of bullying and burnout with suicidal ideation. We presume that the study findings will be useful in establishing evidence on the associated factors of suicidal ideation in the context of Bangladesh.

## Methods

### Study design and setting

We conducted a cross-sectional study in Bangladesh between February 26, 2021, and July 10, 2021, among nurses who were working in clinical settings. Nurses were recruited from all over Bangladesh using social media platforms and from eight tertiary hospitals in the two largest divisions (such as Dhaka and Sylhet). To be employed and work as registered nurses in Bangladesh, nurses have to achieve a diploma (3-year program) degree or a bachelor’s (4-year program) degree^33^. We recruited the nurses if they were registered with the Bangladesh Nursing and Midwifery Council (BNMC) and had at least one year of working experience.

### Study variables

The outcome variable of interest was suicidal ideation, and the exposure variables were workplace bullying and burnout. The covariates include demographic variables (sex, age, marital status, educational level, monthly salary, and smoking status) and occupational variables (type of job, level of hospital, the administrative division of workplace, work department, weekly working hours, years of experience, and had sufficient equipment to manage patients).

### Measurement tools

#### Workplace bullying

Workplace bullying (WPB) was measured using a 9-item Short Negative Acts Questionnaire (S-NAQ-9). The items include a range of negative behaviours that covers both aggravation and victimization aspects that provides a comprehensive assessment of workplace bullying dynamics^34^. The nine questions pertain to whether a person was bullied in the preceding six months. The responses to the items ranged from 1 to 5, with 1 indicating “never” and 5 denoting “daily”. The overall score of the tool ranged from 9 to 45, with a higher score indicating more bullying. Respondents who scored below 15 can be considered “non-exposed” to workplace bullying, while those who scored between 15 and 22 were at “high risk” of becoming victims of bullying or may already be immersed, and who scored above 23 can be considered “targeted” of workplace bullying^23^. Previous numerous studies utilized this tool to measure bullying in several countries in workplace contexts^35,36^. In our study, the McDonald’s omega of the tool was calculated at 0.88, which demonstrates the excellent internal consistency of the tool.

#### Burnout

In this study, the Burnout Measure-Short version (BMS-10) was applied to measure burnout^37^. This scale investigates a person's physical, emotional, and mental well-being by inquiring about 10 items on the main components of burnout thought. A seven-point Likert scale, ranging from 1 (never) to 7 (always), is utilized to score each item. The possible responses for the 10 items ranged from 10 to 70. The response values were divided by 10 to determine a person's overall burnout score. Finally, the overall burnout scores ranged from 1 to 7. To determine burnout, the overall score was classified based on a cut-off point of 4, and those who experienced it received an overall score of 4. The BMS-10 was widely applied among nurses^38,39^. In this study, the McDonald's omega of the BMS-10 was shown to be 0.89.

#### Suicidal ideation

Suicidal ideation was measured by using the Suicidal Ideation Attributes Scale (SIDAS-5)^40^. Only for item-2, with controllability being reverse-scored (10 = 0, 9 = 1,..…,1 = 9, 0 = 10), and the overall SIDAS scores are determined as the sum of the five items (including reverse-scored item-2), with total scale scores ranging from 0 to 50. A cut-off of 21 on the SIDAS-5 signifies a significant risk of suicidal ideation. The level of suicidal ideation were categorized as no ideation (score 0), low ideation (score 1–20), and high ideation (score 21–50). This scale showed excellent reliability among adults^41^. In this study, the McDonald's omega of the SIDAS-5 was found to be 0.72.

#### Questionnaire for data collection

Based on a review of the literature, a structured questionnaire was created. Two senior clinical nurses and two public health specialists from Bangladesh reviewed the initial questionnaire. We further revised the questionnaire in response to their comments. We piloted the questionnaire among 20 nurses before data collection; however, in the final analysis, we did not include them. We listed the study's goal and objective on the questionnaire's front page, along with an option for informed consent. On the second page, we asked the respondents’ demographic information, and on the third page, we asked their occupational information. The questions about S-NAQ-9, BMS-10, and SIDAS-5 were asked on the subsequent pages.

#### Data collection

**Figure 1:**
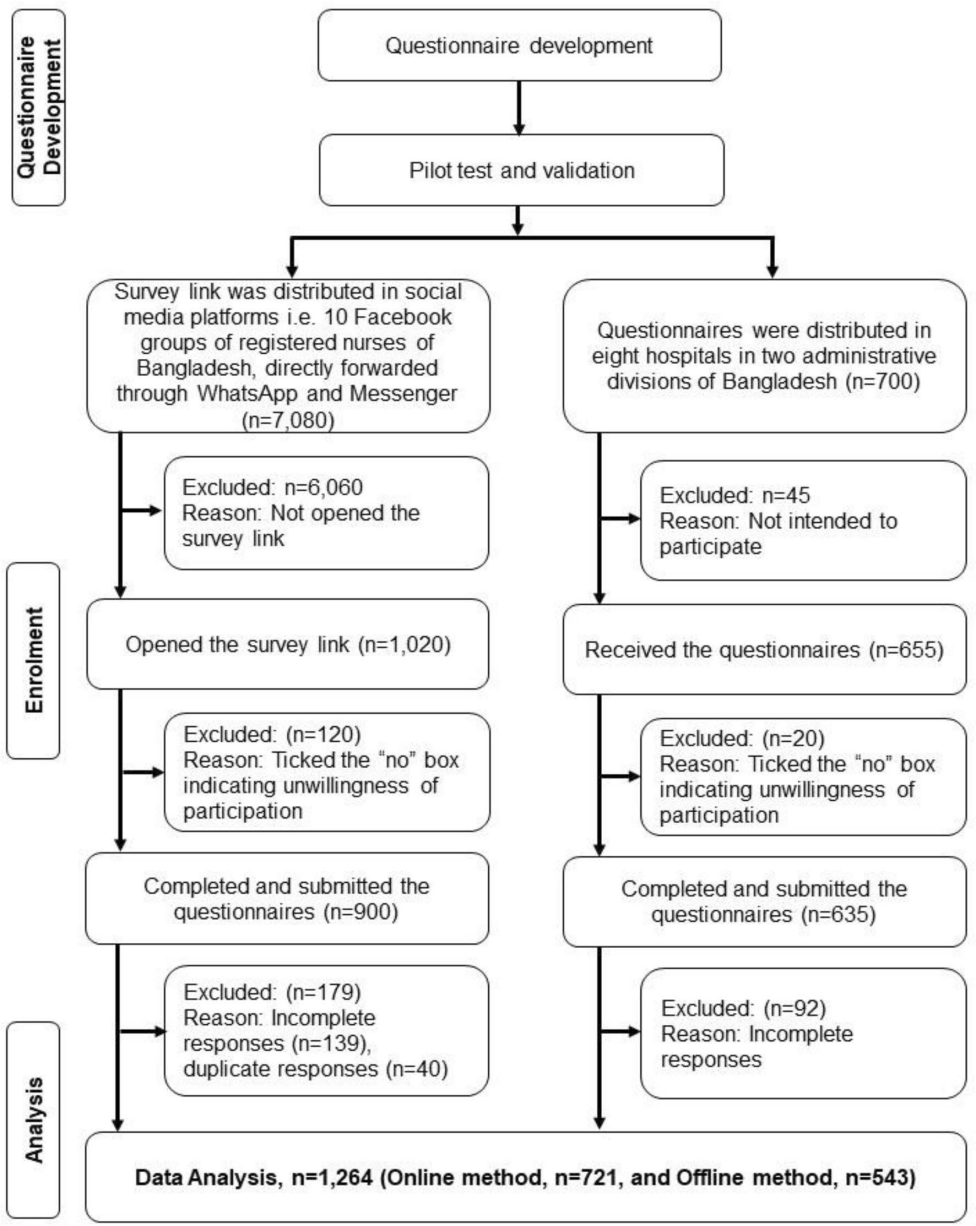
Flowchart of the sample recruitment process.

Face-to-face interview and data collection from the healthcare workers like physicians or nurses during the Coronavirus disease-19 (COVID-19) pandemic required a lot of extra precautions. We used a convenient sampling method for data collection. Considering the potential barriers to face-to-face data collection from the health care workers, we requested using the social platform the registered nurses in Bangladesh to participate in this study^32^. Firstly, the structured questionnaire was transformed into “Google Form,” and an online link was generated. By utilizing the available social media platforms (“WhatsApp, Messenger, and Facebook”), we requested the nurses for their participation after sending them the online link to the questionnaire. The participants were requested to provide only one response. We identified the participants by their demographic characteristics if anyone provided any duplicate response and they were excluded. In return, 721 completed responses were gathered using this technique, and the responses were automatically inputted into a spreadsheet of excel. Secondly, to obtain the optimal sample size, we further distributed 700 printed questionnaires among the clinical nurses who did not participate in the online survey in conveniently chosen eight tertiary hospitals in two major divisions in Bangladesh (“Dhaka and Sylhet”). We provided them with 7 days to fill out the questionnaire, and after the given time, the data collectors collected completed questionnaires (655 copies). After excluding the questionnaire with missing data, we manually entered 543 completed responses into the primary dataset. Finally, by applying two of these approaches of data collection, 1264 completed responses were collected and analyzed in this study. The whole sample recruitment process is presented in Fig. 1, which was published elsewhere in an article from the same project^42^.

#### Data analysis

We used the statistical software STATA-16 for data analysis. We drew the country map by R 4.2.2, and the Directed Acyclic Graph (DAG) by DAGitty, a web-based environment for drawing, formatting, and analyzing cause and effect illustrations^43^. The data were checked and cleaned in an Excel spreadsheet before formal analysis. In this study, data were analyzed using both descriptive and inferential statistics. For categorical data, frequency distribution and percentages were performed as descriptive statistics. Similarly, for continuous variables, mean, and standard deviation were calculated. In terms of inferential statistics, chi-square tests and a Poisson regression model with robust error variance were incorporated. The unadjusted association was investigated using the chi-square test between high suicidal ideation and the explanatory variables. The Poisson regression model was fitted to investigate the adjusted association between suicidal ideations and the explanatory variables that were significant in the chi-square (unadjusted) test at a priori-specified *p* value of 0.1, including sex (as a potential confounder). The Poisson regression model with a robust error variance, also known as modified Poisson regression, was utilized previously as an alternative logistic regression model if the prevalence of binary outcome is more than > 10%^44,45^, where our prevalence was 13.61%. The Poisson regression with a robust error variance in cross-sectional studies demonstrated in reducing the overestimation of the association^46^. Secondly, in the case of convergence failure with the log-binomial model, modified Poisson regression with a robust error variance performs better in estimating the prevalence ratio from a cross-sectional study^47^. The Poisson regression model produces prevalence ratio (PR) which is also considered as relative risk (RR), as we reported the findings of our study^48^. In the model, we also controlled the interaction effects of workplace bullying and burnout on suicidal ideation. The Directed Acyclic Graph (DAG) was created for the evaluation of covariates selection in the analysis of the effects of workplace bullying on suicidal ideation. Workplace bullying is exposure, and suicidal ideation is the outcome.

#### Ethical issue

The goals and objectives of the study were clearly stated on the front page of the questionnaire. As a result, prior to participating, the participants were well-informed about the research and gave their informed consent. The respondents were allowed to withdraw at any moment, and the investigators also ensured that the data of the participants would remain anonymous. This study adhered to the ethical standards outlined in the 2013 Helsinki Declaration for medical research with human beings^49^. We developed a protocol before conducting the study, where the methodology of the study was outlined. The protocol was submitted to the ethics committee of “Begum Rabeya Khatun Chowdhury Nursing College Sylhet, Bangladesh”, for reviewing and approval, and it provided the ethical clearance (ID: BRKCNC/IRB/2021/5).

## Results

### Demographic and occupational characteristics of participants

In Table 1, the demographic and occupational characteristics of participants are presented. Among the study participants, female was 70.02%, and the mean of age was 28.41 (SD = 5.54) years. Almost half (53.40%) were married, and 90.90% were never smokers. Of the participants, 59.81% engaged in government jobs, and 71.99% worked at tertiary-level hospitals. One-third (33.62%) of them had work experience of 6 years and more. And over half of them (52.93%) lacked the necessary equipment to manage patients.

**Table 1:** Demographic and occupational characteristics of participants (n = 1264).

	n	Percent (%)/mean (SD)
*Demographic variables*		
Sex		
Male	379	29.98
Female	885	70.02
Mean age (year)		28.41 (5.54)
Age group (year)		
< 25	303	23.97
25–29	604	47.78
≥ 30	357	28.24
Marital status		
Unmarried	589	46.60
Married	675	53.40
Educational level		
Diploma degree	520	41.14
Bachelor’s degree	514	40.66
Master’s degree or above	230	18.20
Monthly salary		
< 21,000 BDT	361	28.86
21,000–29,999 BDT	513	41.01
≥ 30,000 BDT	377	30.14
Smoking status		
Never smoker	1149	90.90
Past smoker	54	4.27
Current smoker	61	4.83
*Occupational variables*		
Type of job		
Government	756	59.81
Private	508	40.19
Level of hospital		
Primary	147	11.63
Secondary	207	16.38
Tertiary	910	71.99
Administrative division of workplace		
Dhaka	618	48.89
Chattagram	132	10.44
Sylhet	375	29.67
Rajshahi	34	2.69
Khulna	29	2.29
Barishal	24	1.90
Rangpur	31	2.45
Mymensingh	21	1.66
Work department		
Critical ward	320	25.32
Emergency	80	6.33
General ward	197	15.59
Gynecological ward	97	7.67
Medicine ward	326	25.79
Surgery ward	244	19.30
Weekly working hours		
≤ 36 h	597	47.34
37–48 h	520	41.24
> 48 h	144	11.42
Years of experience		
< 3 years	435	34.41
3–5 years	404	31.96
≥ 6 years	425	33.62
Had sufficient equipment to manage patients		
Yes	595	47.07
No	669	52.93

*SD* standard deviation, *n* number.

### Workplace bullying, burnout, and suicidal ideation characteristics of participants

**Figure 2:**
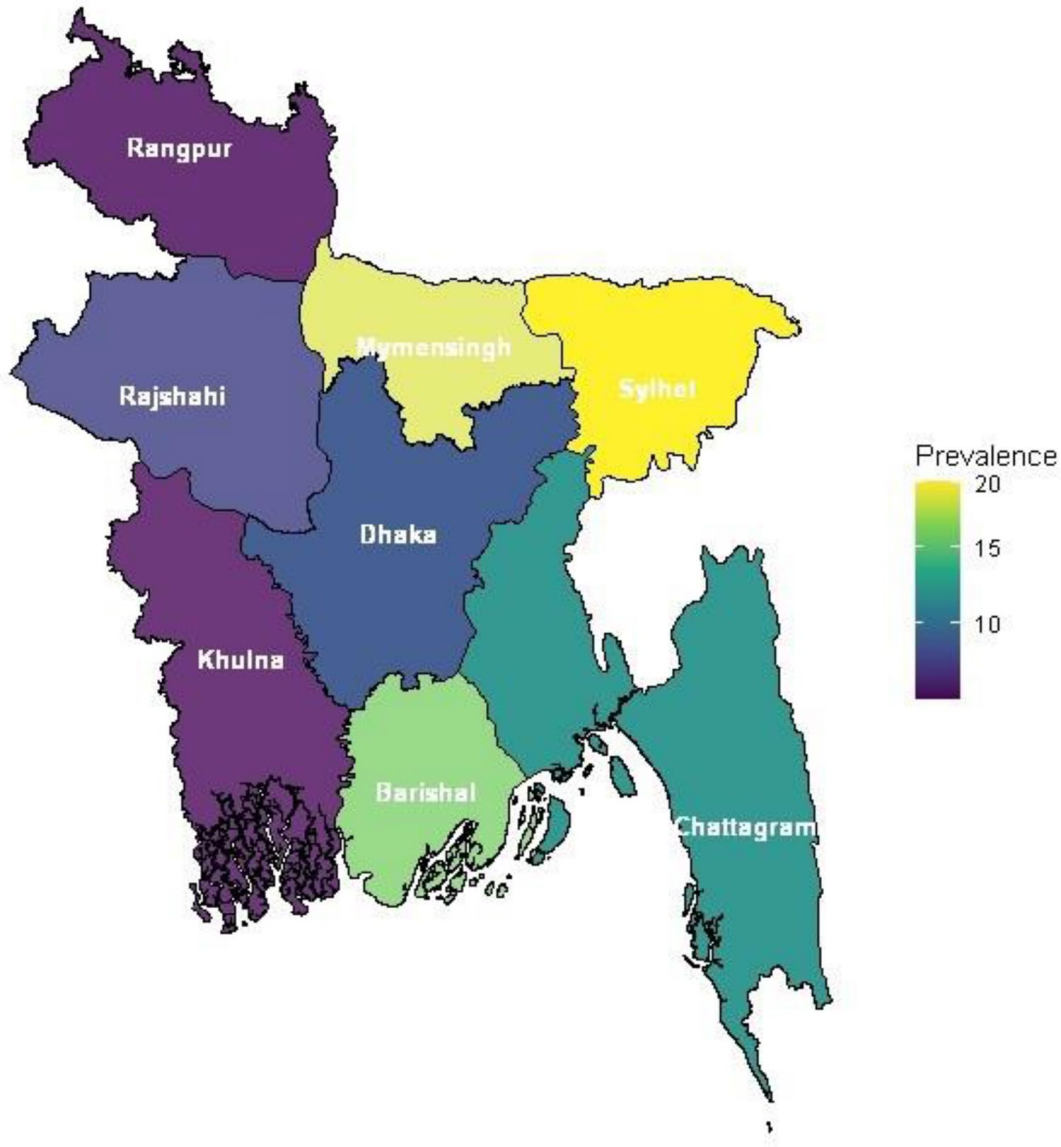
Distribution of the prevalence of suicidal ideation across eight divisions of Bangladesh. This figure was constructed in the statistical program R 4.1.3. by using cartography, sf, maps, and ggplots2 packages (https://cran.r-project.org/web/packages/). The prevalence of suicidal ideation is illustrated in eight division of Bangladesh that is distinguished by several colours.

In Table 2, the workplace bullying, burnout, and suicidal ideation characteristics of participants are presented. More than one-third of the nurses were at high risk (36.68%) of bullying and 19.30% were targeted to bullying, and burnout was found among 21.45% of nurses. Almost half of them had low (49.37%), and 13.26% had high levels of suicidal ideation. The distribution of the prevalence of suicidal ideation across the eight divisions of Bangladesh is presented in Fig. 2.

**Table 2:** Distribution of workplace bullying, burnout, and suicidal ideation of participants (n = 1264).

Variables	n	Percent (%)
Workplace bullying (n = 1254)		
Non-exposed	483	38.21
High risk	529	41.85
Targeted	252	19.94
Burnout		
No	985	78.55
Yes	269	21.45
Suicidal ideation		
No ideation	468	37.17
Low ideation	624	49.56
High ideation	167	13.26

### Unadjusted association of high suicidal ideation with workplace bullying, burnout, and other study variables

In Table 3, the unadjusted association of high levels of suicidal ideation with workplace bullying, burnout, and other study variables is presented. The suicidal ideation (high level) was found to be the highest (26.80%, *p* < 0.001) among the targeted to bullying nurses. The nurses who were exposed to burnout had a significantly higher level of suicidal ideation (23.42%, *p* < 0.001) than the non-burnout nurses. The eldest age group (≥ 30 years) had significantly higher (20.17%, *p* < 0.001) suicidal ideation. Whose monthly income was highest (≥ 30,000 BDT (Bangladeshi Taka)), their suicidal ideation was also found to be highest (18.97%, *p* < 0.001). Suicidal ideation was higher among government jobholder nurses (16.40%, *p* < 0.001) than among private job holders. Suicidal ideation was found to be the highest in nurses working in the Sylhet division (20.27%, *p* < 0.001). Among emergency department nurses, suicidal ideation was found to be the highest (31.25%, *p* < 0.001). Nurses with the most work experience (6 years) had the highest rate of suicidal ideation. For those who did not have sufficient equipment to manage patients in their workplace, their suicidal ideation was found to be highest (17.27%, *p* < 0.001).

**Table 3:** Unadjusted association of high suicidal ideation with workplace bullying, burnout, and other variables (n = 1264).

Variables	High ideation	Low/no ideation	χ^2^	*p* value
	n (%)	n (%)		
Workplace bullying				
Non-exposed	13 (2.70)	468 (97.30)	91.19	**< 0.001**
High risk	87 (16.48)	441 (83.52)		
Targeted	67 (26.80)	183 (73.20)		
Burnout				
No	102 (10.39)	880 (89.61)	31.33	**< 0.001**
Yes	63 (23.42)	206 (76.58)		
*Demographic variables*				
Age, years				
< 25	31 (10.30)	270 (89.70)	20.66	**< 0.001**
25–29	64 (10.65)	537 (89.35)		
≥ 30	72 (20.17)	285 (79.83)		
Sex				
Male	46 (12.27)	329 (87.73)	0.46	0.497
Female	121 (13.69)	763 (86.31)		
Educational degree				
Masters or above	33 (14.35)	197 (85.65)	1.33	0.515
Bachelor	61 (11.94)	450 (88.06)		
Diploma	73 (14.09)	445 (85.91)		
Marital status				
Married	94 (13.95)	580 (86.05)	0.59	0.444
Unmarried	73 (12.48)	512 (87.52)		
Monthly income				
< 21,000 BDT	26 (7.30)	330 (92.70)	20.25	**< 0.001**
21,000–29,999 BDT	69 (13.45)	444 (86.55)		
≥ 30,000 BDT	70 (18.97)	307 (81.43)		
Smoking status				
Never smoker	147 (12.85)	997 (87.15)	5.36	0.068
Past smoker	6 (11.11)	48 (88.89)		
Current smoker	14 (22.95)	48 (88.89)		
*Occupational variables*				
Type of job				
Government	124 (16.40)	632 (83.60)	16.19	**< 0.001**
Private	43 (8.55)	460 (91.45)		
Level of hospital				
Tertiary	135 (14.90)	771 (85.10)	9.73	**0.008**
Secondary	14 (6.80)	192 (93.20)		
Primary	18 (12.24)	129 (87.76)		
Administrative division of workplace				
Dhaka	61 (9.87)	557 (90.13)	22.86	**< 0.001**
Chattagram	16 (12.12)	116 (87.88)		
Sylhet	75 (20.27)	295 (79.73)		
Others^a^	15 (10.79)	124 (89.21)		
Work department				
Critical ward	39 (12.19)	281 (87.81)	27.83	**< 0.001**
Emergency	25 (31.25)	55 (68.75)		
General ward	25 (12.69)	172 (87.31)		
Gynecological ward	6 (6.19)	91 (93.81)		
Medicine ward	44 (13.71)	277 (86.29)		
Surgery ward	28 (11.48)	216 (88.52)		
Weekly working hours				
≤ 36 h	88 (14.79)	507 (85.21)	2.24	0.327
37–48 h	61 (11.80)	456 (88.20)		
> 48 h	18 (12.50)	126 (87.50)		
Years of experience				
< 3 years	40 (9.26)	392 (90.74)	11.17	**0.004**
3–5 years	55 (13.65)	348 (86.35)		
≥ 6 years	72 (16.98)	352 (83.02)		
Had sufficient equipment to manage patients				
Yes	52 (8.77)	541 (91.23)	19.69	**< 0.001**
No	115 (17.27)	551 (82.73)		

^a^Others = Rajshahi, Khulna, Barishal, Rangpur, and Mymensingh.

*n* number, *BDT* Bangladeshi Taka.

Significant values are in [bold].

### Poisson regression model with a robust error variance to find the adjusted association of high suicidal ideation with workplace bullying, burnout, and other study variables.

**Figure 3:**
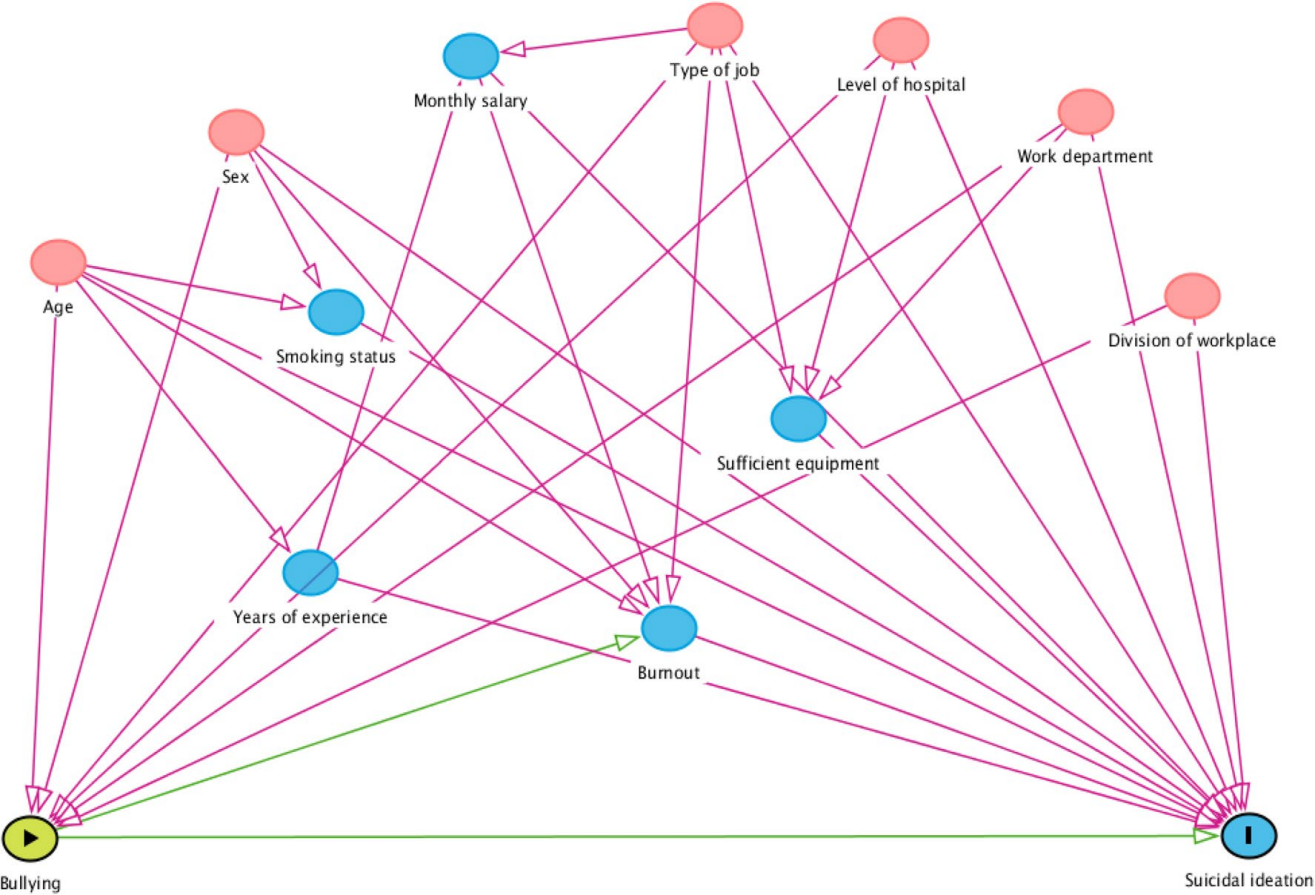
Directed acyclic graph (DAG) for evaluation of covariates selection in the analysis of effects of workplace bullying on suicidal ideation. Workplace bullying is exposure, and suicidal ideation is the outcome. This figure was constructed through DAGitty (http://www.dagitty.net). The green node with a black border represents the exposure variable, and the blue node with a black border represents the outcome variable. Red nodes represent the confounding variables. The blue nodes represent the ancestor of the outcome variable. The red paths are the backdoor/biasing paths, and the green paths are the causal paths.

The Directed Acyclic Graph (DAG), which was created for the evaluation of covariates selection in the analysis of the effects of workplace bullying on suicidal ideation, is presented in Fig. 3.

In Table 4, the Poisson regression model represents the adjusted association of high suicidal ideation with workplace bullying, burnout, and other study variables. Nurses exposed to high risk of bullying and targeted to bullying were 6.22 times (RR = 6.22, 95% CI 3.13–12.38) and 7.61 times (RR = 7.61, 95% CI 3.53–12.23) more likely to have a high level of suicidal ideation than those who were non-exposed. Similarly, the burnout-exposed nurses were significantly 8.95 times (RR = 8.95, 95% CI 2.84–28.20) more likely to have higher suicide ideation than non-burnout nurses. The interaction effects of high-risk bullying (RR = 0.12, 95% CI 0.03–0.41) and targeted bullying (RR = 0.21, 95% CI 0.06–0.70) along with burnout significantly predicted suicidal ideation. Suicidal ideation was significantly higher in the middle (RR = 1.81, 95% CI 1.04–3.13) and higher income groups (RR = 2.04, 95% CI 1.12–3.72). Suicidal ideation was significantly higher among the nurses from the Chattagram division (RR = 2.52, 95% CI 1.82–3.49) than the nurses in Dhaka. Emergency ward nurses had 2.43 times (RR = 2.43, 95% CI 1.53–3.84) more suicidal ideation compared to general ward nurses. Finally, nurses who did not have enough equipment to manage patients had significantly higher (RR = 1.50, 95% CI 1.10–2.04) suicidal ideation than those who had. Additionally, a subgroup analysis was conducted using two distinct methods for sample recruitment: online and offline. Detailed information can be found in Supplementary file-2.

**Table 4:** Poisson regression model with robust error variance to find the adjusted association of high suicidal ideation with workplace bullying, burnout, and other variables.

Variables	High suicidal ideation		
	RR	95% CI	*p* value
Workplace bullying			
Non-exposed	Reference		
High risk	6.22	3.13–12.38	**< 0.001**
Targeted	7.61	3.53–16.38	**< 0.001**
Burnout			
No	Reference		
Yes	8.95	2.84–28.20	**< 0.001**
*Interaction effects*			
Workplace bullying × Burnout			
High risk bullying × Burnout (Yes)	0.12	0.03–0.41	**0.001**
Targeted bullying × Burnout (Yes)	0.21	0.06–0.70	**0.012**
*Demographic variables*			
Age, years			
< 25	Reference		
25–29	0.68	0.40–1.14	0.146
≥ 30	0.93	0.50–1.75	0.831
Sex			
Male	Reference		
Female	1.49	1.01–2.19	**0.045**
Monthly income			
< 21,000 BDT	Reference		
21,000–29,999 BDT	1.81	1.04–3.13	**0.035**
≥ 30,000 BDT	2.04	1.12–3.72	**0.020**
Smoking status			
Never smoker	Reference		
Past smoker	1.59	0.70–3.58	0.266
Current smoker	1.70	0.96–3.00	0.069
*Occupational variables*			
Type of job			
Government	Reference		
Private	1.00	0.59–1.70	0.997
Level of hospital			
Tertiary	Reference		
Secondary	0.63	0.36–1.12	0.116
Primary	1.02	0.61–1.72	0.936
Administrative division of workplace			
Dhaka	Reference		
Chattagram	1.22	0.68–2.18	0.514
Sylhet	2.52	1.82–3.49	**< 0.001**
Others^a^	1.26	0.71–2.25	0.427
Work department			
General ward	Reference		
Critical ward	1.00	0.61–1.63	0.999
Emergency	2.43	1.53–3.84	**< 0.001**
Gynecological ward	0.48	0.20–1.12	0.089
Medicine ward	1.22	0.77–1.94	0.392
Surgery ward	0.95	0.57–1.58	0.843
Years of experience			
< 3 years	Reference		
3–5 years	1.09	0.71–1.66	0.693
≥ 6 years	0.81	0.49–1.33	0.406
Had sufficient equipment to manage patients			
Yes	Reference		
No	1.50	1.10–2.04	**0.010**

^a^Others = Rajshahi, Khulna, Barishal, Rangpur, and Mymensingh.

*n* number, *RR* relative risk, *CI* confidence interval, *BDT* Bangladeshi Taka.

Significant values are in [bold].

## Discussion

The aim of the current study was to investigate the association of workplace bullying and burnout with suicidal ideation among Bangladeshi nurses and also identify the associated demographic and occupational factors of suicidal ideation. We found that bullying and burnout were significantly associated with suicidal ideation in our adjusted model. Our model also showed that monthly income, geographical location of the workplace, department of work, and insufficiency of equipment to manage patients were also significantly associated with suicidal ideation. We presented the direction of the association between suicidal ideation and other predictors, as well as illustrated the prevalence across the country by it’s geographical divisions.

The incidence of bullying against nurses was reported to range from 17 to 76% globally^13,50^. We found 36.86% of the nurses were at high risk of bullying, and 19.30% were targeted to bullying^51^. The prevalence of bullying was found to be significantly higher, whereas the prevalence in Norway was only 8.6%, which seems to be half of our study^52^. The bullying was reported to be higher among the other healthcare workers, and their suicidal ideation was also reported to be associated with consistent exposure to bullying. Such as Shabazz et al.^53^ found 44% of obstetrics and gynecology doctors reported being bullied or undermined on a consistent basis in the UK. We found suicidal ideation was 5 times more among the nurses who were at high-risk and targeted bullying than the nurses who were non-exposed. Similar to our findings, bullying was reported as a predictor of suicidal ideation among nurses in several previous study^15^. Wall et al.^54^ found a higher rate of suicide ideation among Italy and Swedish healthcare workers who experienced harassment or denigration at workplace. Similarly, bullying at the workplace is associated with serious suicide ideation, according to a study conducted by Sterud et al.^55^ among Norwegian ambulance workers. Bullying and suicidal ideation were associated among the general people as well—a systematic review reported a significant association between bullying and suicidal ideation, and suicidal behavior^56^.

We found 21.36% of nurses experienced burnout, 10.60% had severe, and 4.59% needed professional assistance due to burnout. We also found that suicidal ideation was 2 times higher among the nurses who reported to be burnout. Similar to our finding, the association between burnout and suicidal ideation among nurses was established by previous study as well^21^. According to a study in Taiwan, nurses with burnout had higher suicidal ideation, which accounted for 19.4% and 8.6% in the case of personal burnout and burnout related to client^27^. Among nurses in German, 21.7% had suicidal ideation, and they reported an approximate prevalence of suicidal ideation that we found in this study due to burnout^57^. As long as the nurses, burnout and suicidal ideation were observed among the other healthcare workers as well, and the association was reported among them. According to Dyrbye et al.^58^, 10% of medical school students reported suicide ideation, while almost 50% of them reported burnout. They concluded that there was a significant positive association between the two and advised lowering the level of burnout in order to prevent student suicide.

Regarding geographical location, nurses in the Sylhet division reported having higher suicide thoughts than those in Dhaka. Similar to our study, suicidal ideation among Canadian nurses was significantly varied by regional location found in research conducted by Stelnicki et al.^59^, where data from 3969 nurses were analyzed. Yenilmez et al.^60^ conducted a study in Turkey among the general population and found a significant association between geographical variation and suicidal ideation. Individuals living at nursing homes in the north and midwest of the United States as well as in Maine, Vermont, and New Hampshire, had consistently higher suicide ideation prevalence than other states, according to a nationally representative study^61^.

In this study, nurses who worked in emergency departments had 2.27 times more suicidal ideation than those who worked in routine wards. It is reported that emergency department nurses experience higher levels of suicidal ideation^62^. This issue may be explained as a slightly higher occupational hazard as they consistently have different types of patients for a certain time. Previous studies also reported that emergency department nurses frequently faced more aggressive and violent behavior from patients and their relatives^63,64^. However, Ariapooran et al.^65^ investigated nurses' anxiety and suicidal ideation in Malayer, Iran, during the COVID-19 outbreak, but they found no association between departmental variation and suicidal ideation—even though emergency department nurses were significantly more anxious than other departments’ nurses.

The role of economic inequalities in suicidal ideation was discussed previously by Zeng et al.^66^. A study conducted by Ishikawa et al.^67^ in Japan found that low income was a potential predictor of suicidal ideation among healthcare professionals. In Bangladesh, the starting salary is still disappointing; in public settings, it ranges from 26,000 BDT to 28,000 BDT, which may be exceedingly low to survive, despite the fact that the scenario is more complicated for nurses who work in private hospitals, where the least salary can start from 16,000 BDT^68^. As a consequence, Akter et al. (2019) addressed that low earnings even impacted the Bangladeshi nurses’ quality of life and also posed a potential barrier to their career progress^68^. Our findings indicate that nurses in the higher and middle-income categories showed higher levels of suicidal ideation compared to those in the lower-income category. This can be explained by the work experience of the nurses. We found that nurses with higher work experience showed a higher prevalence of suicidal ideation. In the context of Bangladesh, nurses have a minimum opportunity to get promoted in their work position, get recognition, and get an increment in their salary along with their work experience^14^. This may pose psychological stress among nurses, which may lead to suicidal ideation. Though experienced nurses get higher salaries than novice nurses, the salary is still minimal, which could be a reason for their high suicidal ideation. While nurses' salaries are fixed, the opportunity to earn additional income through overtime work may reduce their financial concerns. However, this can also lead to increased stress and mental health issues, potentially contributing to suicidal ideation. Addressing this issue requires further research in a specific attention in Bangladesh.

We found suicidal ideation was significantly higher among those who lacked the necessary workplace equipment to care for patients. If the workplace is not well equipped, providing quality care becomes jeopardized for the nurses, and the patient's life may even be in danger. Therefore, to provide better care during their busy schedules, healthcare workers may experience incremental stress due to insufficient equipment and become violence victims^69^. Consequently, equipment support can be buffered by other facility-level issues, which may influence nurses' suicidal ideation. A safe workplace with the required instrumental support, as well as certain efforts to enhance the working environment, may improve nurses' mental health and assist in preventing suicide ideation.

## Strengths and limitations

This study has certain strengths and limitations. Suicidal ideation among Bangladeshi nurses was not investigated priorly, which can be considered a potential strength of our study. We recruited a large sample size across all divisions of the country, which may provide optimum statistical power for our analysis. For the purpose of this study, a convenient sampling approach was employed, utilising two distinct recruitment methods: online and in-person interviews. The utilisation of in-person interviews as a research method has proven effective in mitigating response bias. Additionally, when applied to a sizable sample, this approach enhances the likelihood of arriving at a valid and reliable conclusion. Despite the strengths, our study has some limitations as well. Although we recruited a large sample size, the sampling technique was not random; thus, selection bias may have occurred due to the convenient sampling procedure. We were not able to recruit an equal number of participants from the eight divisions of the country. We used a self-reported questionnaire for data collection at the time of the COVID-19 pandemic; therefore, reporting bias could be unavoidable. As the nature of the cross-sectional study, the evidence can not be considered a causal association. It is recommended that future research be conducted to evaluate the association between harassment, bullying and burnout taking into account existing policies, levels, and other relevant factors within the healthcare setting.

## Conclusion

We found a significant association of workplace bullying and burnout with suicidal ideation among nurses in Bangladesh. Occupational factors like monthly income, working location, working department, and lack of necessary instruments to serve patients were also associated with higher suicidal ideation. These findings can assist policymakers and healthcare authorities in initiating an effective strategy for preventing suicidal thoughts among nurses. The high prevalence of suicidal ideation demonstrates that a safe workplace and initiatives are necessary to enhance nurses' overall mental health, which may interact with their suicidal ideation. As suicide is becoming a global issue, preventive measures should be scaled-up. Ensuring a safe workplace with necessary instrumental support may improve the working environment and reduce suicidal ideation. We suggest further studies with large sample sizes utilizing a random approach and focusing on a wide variety of factors, including workplace environment and safety issues that are related to the nurses’ suicidal ideation. Further research on investigating how bullying, burnout, and suicidal ideation impact comprehensive patient care is also recommended.

### Supplementary Information

Supplementary Tables.

Supplementary Information 1.

## Data Availability

Data of the study is provided as Supplementary file-2.
