# Supplementary material for: Association of workplace bullying and burnout with nurses’ suicidal ideation in Bangladesh
Source: Sci Rep. 2023 Sep 5;13:14641. doi: 10.1038/s41598-023-41594-4 (PMC10480219; doi:10.1038/s41598-023-41594-4)
Supplement: Supplementary file 1 — Supplementary Tables. [file 41598_2023_41594_MOESM1_ESM.docx]

**Table S1. Poisson regression model with robust error variance to find the adjusted association of high suicidal ideation with workplace bullying, burnout, and other variables** **(online data collection)**

| **Variables** | **High suicidal ideation** | | |
| --- | --- | --- | --- |
|  | **RR** | **95% CI** | ***p*-value** |
| **Workplace bullying** |  |  |  |
| Non-exposed | Reference |  |  |
| High risk | 5.29 | 1.41-19.82 | **0.013** |
| Targeted | 3.62 | 0.35-37.03 | 0.278 |
| **Burnout** |  |  |  |
| No | Reference |  |  |
| Yes | 6.17 | 1.60-23.76 | **0.008** |
| **Interaction effects** |  |  |  |
| **Workplace bullying × Burnout** |  |  |  |
| High risk bullying × Burnout (Yes) | 0.24 | 0.05-1.13 | 0.070 |
| Targeted bullying × Burnout (Yes) | 0.92 | 0.08-10.76 | 0.950 |
| **Demographic variables** | | | |
| **Age, years** |  |  |  |
| < 25 | Reference |  |  |
| 25 - 29 | 1.24 | 0.75-2.07 | 0.399 |
| ≥ 30 | 1.98 | 0.90-4.38 | 0.090 |
| **Sex** |  |  |  |
| Male | Reference |  |  |
| Female | 1.98 | 1.01-3.85 | **0.046** |
| **Monthly income** |  |  |  |
| <21,000 BDT | Reference |  |  |
| 21,000-29,999 BDT | 1.21 | 0.69-2.12 | 0.497 |
| ≥30,000 BDT | 1.22 | 0.63-2.37 | 0.554 |
| **Smoking status** |  |  |  |
| Never smoker | 0.30 | 0.12-0.74 | 0.008 |
| Past smoker | 0.72 | 0.27-1.93 | 0.518 |
| **Occupational variables** | | | |
| **Type of job** |  |  |  |
| Government | Reference |  |  |
| Private | 2.01 | 0.98-4.12 | 0.056 |
| **Level of hospital** |  |  |  |
| Secondary | 0.61 | 0.30-1.24 | 0.170 |
| Tertiary | 1.06 | 0.64-1.74 | 0.826 |
| **Administrative division of workplace** |  |  |  |
| Dhaka | Reference |  |  |
| Sylhet | 1.18 | 0.59-2.35 | 0.643 |
| Chittagong | 1.10 | 0.57-2.15 | 0.776 |
| Others^†^ | 1.14 | 0.62-2.10 | 0.664 |
| **Work department** |  |  |  |
| General ward | Reference |  |  |
| Critical ward | 0.72 | 0.36-1.43 | 0.343 |
| Emergency | 1.32 | 0.52-3.38 | 0.561 |
| Gynecological ward | 0.80 | 0.35-1.82 | 0.594 |
| Medicine ward | 0.49 | 0.25-0.97 | **0.040** |
| Surgery ward | 0.59 | 0.28-1.28 | 0.182 |
| **Years of experience** |  |  |  |
| <3 years | Reference |  |  |
| 3-5 years | 0.76 | 0.45-1.29 | 0.311 |
| ≥6 years | 0.79 | 0.43-1.42 | 0.425 |
| **Had sufficient equipment to manage patients** |  |  |  |
| Yes | Reference |  |  |
| No | 1.31 | 0.84-2.05 | 0.231 |

† Others = Rajshahi, Khulna, Barishal, Rangpur, Mymensingh

n = Number

RR = Relative risk

CI = Confidence interval

BDT = Bangladeshi Taka

**Table S2. Poisson regression model with robust error variance to find the adjusted association of high suicidal ideation with workplace bullying, burnout, and other variables** **(offline data collection)**

| **Variables** | **High suicidal ideation** | | |
| --- | --- | --- | --- |
|  | **RR** | **95% CI** | ***p*-value** |
| **Workplace bullying** |  |  |  |
| Non-exposed | Reference |  |  |
| High risk | 13.20 | 1.69-103.15 | **0.014** |
| Targeted | 5.26 | 0.34-80.40 | 0.233 |
| **Burnout** |  |  |  |
| No | Reference |  |  |
| Yes | 4.90 | 0.27-88.57 | 0.282 |
| **Interaction effects** |  |  |  |
| **Workplace bullying × Burnout** |  |  |  |
| High risk bullying × Burnout (Yes) | 0.32 | 0.02-5.84 | 0.442 |
| Targeted bullying × Burnout (Yes) | 1.52 | 0.05-42.30 | 0.804 |
| **Demographic variables** | | | |
| **Age, years** |  |  |  |
| < 25 | Reference |  |  |
| 25 - 29 | 0.59 | 0.18-1.97 | 0.391 |
| ≥ 30 | 0.67 | 0.18-2.47 | 0.550 |
| **Sex** |  |  |  |
| Male | Reference |  |  |
| Female | 0.80 | 0.50-1.29 | 0.368 |
| **Monthly income** |  |  |  |
| <21,000 BDT | Reference |  |  |
| 21,000-29,999 BDT | 2.50 | 0.10-62.76 | 0.578 |
| ≥30,000 BDT | 2.45 | 0.09-68.57 | 0.598 |
| **Smoking status** |  |  |  |
| Never smoker | 0.86 | 0.48-1.53 | 0.605 |
| Past smoker | 0.39 | 0.07-2.17 | 0.285 |
| **Occupational variables** | | | |
| **Type of job** |  |  |  |
| Government | Reference |  |  |
| Private | 0.09 | 0.01-3.87 | 0.213 |
| **Level of hospital** |  |  |  |
| Secondary | 0.36 | 0.03-4.67 | 0.438 |
| Tertiary | 1.00 | 0.12-8.05 | 0.999 |
| **Administrative division of workplace** |  |  |  |
| Dhaka | Reference |  |  |
| Sylhet | 5.04 | 3.21-7.90 | **<0.001** |
| Chattagram | 1.39 | 0.16-11.66 | 0.764 |
| Others^†^ | 10.27 | 0.70-149.63 | 0.088 |
| **Work department** |  |  |  |
| General ward | Reference |  |  |
| Critical ward | 1.45 | 0.64-3.28 | 0.367 |
| Emergency | 1.34 | 0.49-3.64 | 0.570 |
| Gynecological ward | 1.50 | 0.55-4.06 | 0.424 |
| Medicine ward | 1.28 | 0.57-2.89 | 0.553 |
| Surgery ward | 1.79 | 0.79-4.02 | 0.161 |
| **Years of experience** |  |  |  |
| <3 years | Reference |  |  |
| 3-5 years | 1.30 | 0.49-3.46 | 0.602 |
| ≥6 years | 0.91 | 0.31-2.70 | 0.862 |
| **Had sufficient equipment to manage patients** |  |  |  |
| Yes | Reference |  |  |
| No | 2.03 | 1.23-3.35 | **0.006** |

† Others = Rajshahi, Khulna, Barishal, Rangpur, Mymensingh

n = Number

RR = Relative risk

CI = Confidence interval

BDT = Bangladeshi Taka
